# Chemical fingerprinting and quantitative analysis of a *Panax notoginseng *preparation using HPLC-UV and HPLC-MS

**DOI:** 10.1186/1749-8546-6-9

**Published:** 2011-02-24

**Authors:** Hong Yao, Peiying Shi, Qing Shao, Xiaohui Fan

**Affiliations:** 1 Pharmaceutical Informatics Institute, Zhejiang University, Hangzhou 310058, China

## Abstract

**Background:**

Xuesaitong (XST) injection, consisting of total saponins from *Panax notoginseng*, was widely used for the treatment of cardio- and cerebro-vascular diseases in China. This study develops a simple and global quality evaluation method for the quality control of XST.

**Methods:**

High performance liquid chromatography-ultraviolet detection (HPLC-UV) was used to identify and quantify the chromatographic fingerprints of the XST injection. Characteristic common peaks were identified using HPLC with photo diode array detection/electrospray ionization tandem mass spectrometry (HPLC-PDA/ESI-MS^n^).

**Results:**

Representative fingerprints from ten batches of samples showed 27 'common saponins' all of which were identified and quantified using ten reference saponins.

**Conclusion:**

Chemical fingerprinting and quantitative analysis identified most of the common saponins for the quality control of *P. notoginseng *products such as the XST injection.

## Background

Xuesaitong (XST) injection, consisting of total saponins from *Panax notoginseng *(*Sanqi*), was widely used for the treatment of cardiovascular and cerebrovascular diseases in China. As total saponins (including ginsenosides and notoginsenosides) in the XST injection are its active ingredients, quality control of total saponins in the XST injection is critical for its safety, efficacy and stability. Single or simultaneous determination of main components of the total saponin extracts from *P. notoginseng *using high performance liquid chromatography-ultraviolet detection (HPLC-UV) [1-5], high performance liquid chromatography-evaporative light scattering detection (HPLC-ELSD) [6], high performance liquid chromatography-mass spectroscopy (HPLC-MS) [7-13] have been reported but over half of the total saponins were not quantified in these studies due to the lack of saponin references or poor chromatographic resolution. A comprehensive and systematic quality control of saponin extracts is much needed.

Fingerprint analysis is currently developed for quality control in Chinese medicine [14-26] and has been accepted by the WHO for the assessment of herbal medicines [27]. The State Food and Drug Administration (SFDA) of China requires all herbal medicine-derived injections and related materials to use chromatographic fingerprints [28] in standardization.

This article reports a novel fingerprint analytical method for quality control of the XST injection, which may be applicable to other herbal products. Over the previous studies [1-13], the new method features the following advantages. (1) The representative fingerprints show good chromatographic separation for most of visible peaks in the chromatographic profiles at 203 nm; (2) All main saponins (27 visible peaks in chromatographic profiles) are identifiable using high performance liquid chromatography-photo diode array detection/electrospray ionization tandem mass spectrometry (HPLC-PDA/ESI-MS^n^) technique, ten saponin references or data from literature [8-14].

## Methods

### Materials and reagents

**Figure 1:**
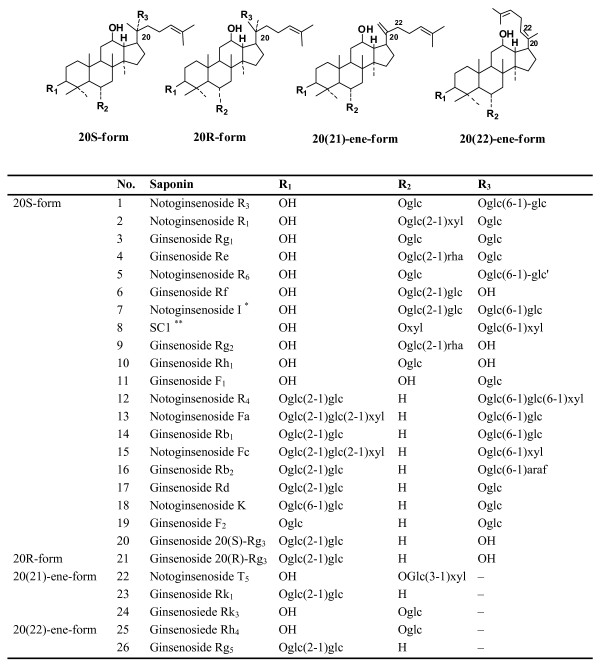
**Structures of the investigated saponins in *P. notoginseng***. glc, β-D-glucose; glc', α-D-glucosexyl, β-D-xylose; rha, α-L-rhamnose; araf, α-L-arabinose (furanose). Notoginsenoside I *, H is instead of OH (C_12_) in 20S-form. SC1 **, 6-O-β-D-xylopyranosyl -20-β-D-xylopyranosyl-(1→6)-β-D-glucopyranosyl dammar-24-ene-3β, 6α, 12β, 20(S)tetraol.

Acetonitrile and methanol (HPLC grade) were purchased from Merck (Darmstadt, Germany). Acetic acid glacial (HPLC grade) was from Tedia (Fairfield, OH, USA). The water used was purified by Milli-Q system (Millipore, USA). Reference compounds, namely notoginsenoside R_1_, ginsenoside Rg_1_, Rg_2_, Rh_1_, Rb_1_, Rb_2_, Rd, Re, 20(S)-Rg_3 _and 20(R)-Rg_3 _were purchased from Jilin University (Shenyang, China). The structures of these compounds are shown in Figure 1. Mixed standard stock solution containing accurately weighed reference compounds was directly prepared in 80% aqueous methanol (v/v). Working standard solutions were prepared by diluting the stock solution with 80% aqueous methanol (v/v) to obtain a series of concentrations for the calibration curves.

### HPLC instrumentationadditional 1 and chromatographic conditions

An Agilent 1100 HPLC system (Agilent Technologies, USA) consisted of a quaternary solvent delivery system, an on-line degasser, an auto-sampler, a column temperature controller and ultraviolet detector coupled with an analytical workstation and an Ultimate™ XB-C_18 _column, 5 μm, 250 mm × 4.6 mm i.d. (Welch Materials, USA) were used in the HPLC-UV experiments. Flow rate was 1.0 ml/min and sample injection volume was 10 μl. Detection wavelength was set at 203 nm and the column temperature was at 30°C. Mobile phase contained deionized water-acetic acid (A; 100:0.01, v/v) and acetonitrile-acetic acid (B; 100:0.01, v/v). The gradient elution was as follows: 19-21.2% B at 0-30 min; 21.2-26% B at 30-35 min; 26-28% B at 35-40 min; 28-38% B at 40-50 min; 38-55% B at 50-60 min; 55% B at 60-65 min; 55-80% B at 65-70 min; 80-95% B at 70-75 min. Re-equilibrium was 10 min; the total run time was 85 min.

### HPLC-MS^n ^instrumentation and chromatographic conditions

Analysis was performed on an Agilent 1100 series LC system equipped with a binary solvent delivery system, an auto-sampler, a column temperature controller, a photo diode array detector and a Finnigan LCQ Deca XP^plus ^ion trap mass spectrometer (Thermo Finnigan, USA) via an ESI interface. The chromatographic conditions were the same for HPLC-UV as described in the previous section. The operating parameters for MS in the negative mode were as follows: collision gas, ultrahigh-purity helium (He); nebulizing gas, high purity nitrogen (N_2_); ion spray voltage, -4.5 kV; sheath gas (N_2_) at a flow rate of 60 arbitrary units; auxiliary gas (N_2_) at a flow rate of 20 arbitrary units; capillary temperature, 350°C; capillary voltage, -15 V; tube lens offset voltage, -30 V. Full scan data acquisition was performed from *m/z *80 to 1800 in MS scan mode. The MS^n ^spectra were obtained with the collision energy for collision-induced dissociation adjusted to 30%-40% of maximum and the isolation width of precursor ions was 2.0Th.

### Sample preparation

Ten samples of the XST injection (Batch No. 20090307, 20090510, 20090310, 20081018, 9042213, 20090312, 20090421, 20090512, 20090504, 20090203), manufactured by three Chinese pharmaceutical companies, were obtained either from pharmacies or factories. For HPLC-PDA-MS^n ^analysis, a certain volume of the injection, according to its nominal content of total saponins, was transferred to a 50 ml volumetric flask and was diluted with 80% aqueous methanol (v/v) to obtain total saponins at a concentration of about 1 mg/ml. For HPLC-UV analysis, the injection was diluted with 80% aqueous methanol (v/v) to obtain total saponins at a concentration of about 0.5 mg/ml. Prior to analysis, the sample solutions were filtered through a 0.45 μm nylon membrane (Whatman, Britain). Spiked injection was produced by mixing sample solutions with the reference solutions at the ratio of 1:1.

### Data analysis

Data analysis was carried out with Similarity Evaluation System for Chromatographic Fingerprint of Traditional Chinese Medicine (version 2004A, National Committee of Pharmacopoeia, China) recommended by the SFDA.

## Results and discussion

### Optimization of HPLC separation

We optimized the separation conditions including the column, mobile phase, detection wavelength, elution gradient and column temperature in this study. Four reversed-phase columns, Agilent Zorbax Eclipse SB-C_18 _columns (250 mm × 4.6 mm, 5 μm; 150 mm × 4.6 mm, 3.5 μm; 100 mm × 2.1 mm, 1.8 μm) and Ultimate™ XB-C_18 _column (250 mm × 4.6 mm, 5 μm) were tested. The results showed that all four columns obtained good peak resolutions in 75 min, 75 min, 45 min and 75 min respectively; however, only two columns with the length of 250 mm (Zorbax Eclipse SB-C_18 _and Ultimate™ XB-C_18_) produced more peaks in chromatograms. Ultimate™ XB-C_18 _column (250 mm × 4.6 mm, 5 μm) was selected in the fingerprint analysis due to its lower cost than Zorbax Eclipse SB-C_18 _column.

The effects of mobile phase composition on chromatographic separation were also studied. The cetonitrile/water system produced more sharp peaks than the methanol/water system; the addition of 0.01% acetic acid in the acetonitrile/water system further improved the peak shape. Moreover, as the retention time of some components such as ginsenoside 20(S)-Rg_3 _and 20(R)-Rg_3 _was long, gradient elution was used in HPLC analysis. Satisfactory separation was achieved in 75 min.

There was no strong absorption for most of saponins in the region of ultraviolet and visible spectra due to their structural characteristics, *eg *lack of conjugation groups in the molecular structures. As the end adsorption wavelength 203 nm is suitable for the assay of ginsenosides and notoginsenosides [1-5], it was selected as the detection wavelength in the experiment. Furthermore, the effects of column temperature on chromatographic separation were also examined. Four column temperatures, namely 20, 25, 30 and 35°C were tested. We found that at 30°C most peaks in chromatography had good resolutions; therefore, 30°C was chosen as the column temperature for the fingerprint analysis.

### HPLC-UV fingerprinting of the XST injection

**Figure 2:**
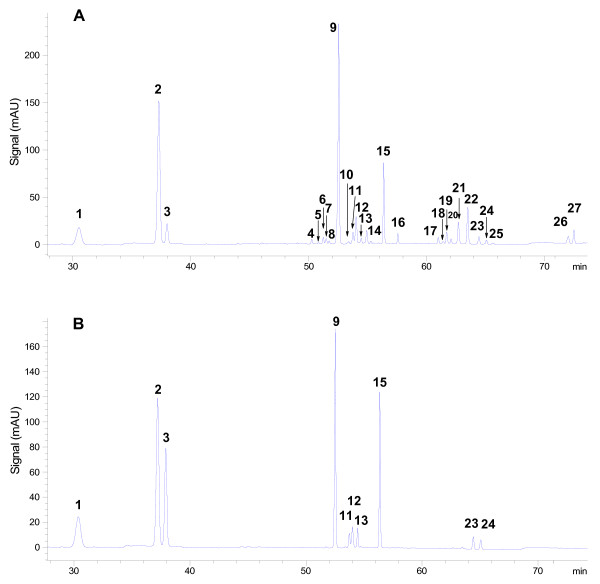
**Chromatograms of (A) the representative fingerprint, (B) mixture standard compounds including (1) notoginsenoside R_1_, (2) ginsenoside Rg_1_, (3) ginsenoside Re, (9) ginsenoside Rb_1_, (11) ginsenoside Rg_2_, (12) ginsenoside Rh_1_, (13) ginsenoside Rb_2_, (15), ginsenoside Rd, (23) ginsenoside 20 (S)-Rg_3 _and (24) ginsenoside 20 (R)-Rg_3_**.

To standardize the fingerprints, we analyzed ten samples using the optimized HPLC-UV method. Peaks found in all ten samples with good resolution were assigned as 'characteristic peaks' and there were 27 characteristic peaks in the fingerprint chromatograms (Figure 2A). The software of Similarity Evaluation System for Chromatographic Fingerprint of Traditional Chinese Medicine was used to evaluate these chromatograms. To exclude the effects of the solvent and baseline fluctuation, we selected the chromatographic data of these ten samples and treated them within the time frame of 28 min to 75 min. The similarities of chromatograms for the ten samples to the reference fingerprints were established using the means of all chromatograms (Additional file 1). The results showed that the ten samples possessed similarities to the reference fingerprints (Additional file 2). While the HPLC-UV fingerprints from different batches and companies varied, the 27 characteristic peaks were common in all samples. Therefore, the detection of these common peaks in HPLC fingerprints is useful in assessing the quality of the XST injection.

### Identification of characteristic peaks

HPLC-PDA/ESI-MS^n ^was used for the components analysis and all 27 characteristic peaks were identified. In the ESI-MS experiment, the molecular weight of each peak was also obtained. By comparing with the ESI-MS^n ^data and HPLC retention time of standard sanponins (Figure 2B and Additional file 3), we identified 10 peaks as notogisenoside R_1_, ginsenoside Rg_1_, Re, Rb_1_, Rg_2_, Rh_1_, Rb_2_, Rd and 20(S)-Rg_3_, 20(R)-Rg_3_. A total of 17 peaks were identified tentatively with the aid of the ESI-MS^n ^data and HPLC retention time of some saponins from previous reports [1-13]. All the identification results are shown in Table 1. In addition, The UV spectra of all peaks in the XST injection were obtained from the PDA chromatogram (Additional file 3). The results showed that among all the peaks in the chromatogram of the XST injection no strong UV absorption within the wavelength range from 210 nm to 400 nm was obtained, suggesting that the XST injection consisted of saponins with few other natural components possessing strong UV absorption, such as flavonoids, lignins, anthraquinones and alkaloids.

**Table 1:** The identification results of saponins in the XST injection by LC/MS^n^

**Peak No**.	Identification	Retention time (min)	**MS[M-H]**^-^	MS data (*m/z*)
1	Notoginsenoside R_1_	34.89	932	799 [M-H-Xyl]^-^; 637 [M-H-Xyl-Glc]^-^; 475 Agl
2	Ginsenoside Rg_1_	39.32	800	637 [M-H-Glc]^-^; 619 [M-H-H_2_O-Glc]^-^; 475 Agl
3	Ginsenoside Re	39.72	945	783 [M-H-Glc]^-^; 637 [M-H-Glc-Rha]^-^; 475 Agl
4	Notoginsenoside R_4_	51.24	1240	1107 [M-H-Xyl]^-^; 1077 [M-H-Glc]]^-^; 945 [M-H-Xyl-Glc; 783 [M-H-Xyl-2Glc]^-^
5	Ginsenoside Rf	51.89	800	637 [M-H-Glc]]^-^; 475 Agl
6	Notoginsenoside Fa	52.17	1240	1107 [M-H-Xyl]^-^; 1077 [M-H-Glc]]^-^; 945 [M-H-Xyl-Glc; 783 [M-H-Xyl-2Glc]^-^
7	Notoginsenoside I	52.39	1092	929[M-H-Glc]^-^; 767 [M-H-2Glc]^-^; 605[M-H-3Glc]^-^
8	SC1	52.56	901	769 [M-H-Xyl]^-^; 637 [M-H-2Xyl]^-^; 475 Agl
9	Ginsenoside Rb1	53.48	1107	945 [M-H-Glc]^-^; 783 [M-H-2Glc]^-^; 621 [M-H-3Glc]^-^; 459 Agl
10	Notoginsenoside Fc	54.32	1209	1077 [M-H-Xyl]^-^; 945 [M-H-2Xyl]^-^; 783 [M-H-2Xyl-Glc]^-^; 621 [M-H-2Xyl-2Glc]^-^; 459 Agl
11	Ginsenoside Rg_2_	54.75	783	637 [M-H-Rha]^-^; 621 [M-H-Glc]^-^; 475 Agl
12	Ginsenoside Rh_1_	55.04	637	475 [M-H-Glc]^-^
13	Ginsenoside Rb_2_	55.30	1077	945[M-H-Arap]^-^; 915[M-H-Glc]^-^; 783[M-HArap-Glc]^-^; 621[M-H-Arap-2Glc]^-^; 459 Agl
14	Ginsenoside F_1_	55.84	637	475 [M-H-Glc]^-^
15	Ginsenoside Rd	57.16	945	783 [M-H-Glc]^-^; 621[M-H-2Glc]^-^; 459Agl
16	Notoginsenoside K	58.32	945	783 [M-H-Glc]^-^; 621[M-H-2Glc]^-^; 459Agl
17	Notoginsenoside T_5_/Unkown	61.70	752	619[M-H-Xyl]^-^; 457 Agl
18	Unkown	62.09	765	603[M-H-Glc]^-^
19	Notoginsenoside T_5_/Unkown	62.42	752	619[M-H-Xyl]^-^; 457 Agl
20	Unkown	62.81	765	603[M-H-Glc]^-^
21	Ginsenoside Rk_3_	63.42	619	551 [M-H-C_5_H_10_]^-^
22	Ginsenoside Rh_4_	64.18	619	551 [M-H-C_5_H_10_]^-^
23	20(S)-ginsenoside Rg_3_	65.14	783	621 [M-H-Glc]^-^; 459 Agl
24	20(R)-ginsenoside Rg_3_	65.86	783	621 [M-H-Glc]^-^; 459 Agl
25	Ginsenoside F_2_	66.05	783	621 [M-H-Glc]^-^; 459 Agl
26	Ginsenoside Rk_1_	72.47	765	603[M-H-Glc]^-^
27	Ginsenoside Rg_5_	72.89	765	603[M-H-Glc]^-^

### Determination of the main saponins in the XST injection

As shown in Figure 2A, 27 saponins were well separated, of which 25 were potentially identified (Table 1). The ratio of total saponin peak area to all peaks (except for solvent peaks and baseline fluctuation in 0-28 min) in the chromatogram of each sample was beyond 95%. Thus, a method for quantification of the 27 saponins should provide a global and systematical evaluation for the quality control of the XST injection. However, it was difficult to obtain the reference compounds for all 27 saponins; we were only able to obtain ten, including notoginsenoside R_1_, ginsenoside Rg_1_, Re, Rb_1_, Rg_2_, Rh_1_, Rb_2_, Rd, 20(S)-Rg_3 _and 20(R)-Rg_3_. Some reports [1-3] found that the slopes of regression equations for most of the determined saponins, such as notoginsenoside R_2_, R_4_, Fa, ginsenoside Rg_1_, Re, Rf, Rb_1_, Rg_2_, Rh_1 _and Rd were approximately negatively correlated to their molecular weights by HPLC-UV at 203 nm (Additional file 4) and that the regression equations of some saponins with similar molecular weights were also close to each other under the same chromatographic condition (Additional file 5, 6, 7, 8 and 9).

Ten saponins, namely R_1_, ginsenoside Rg_1_, Re, Rb_1_, Rg_2_, Rh_1_, Rb_2_, Rd, 20(S)-Rg_3 _and 20(R)-Rg_3 _were quantitatively determined and the rest 17 saponins without standard references were semi-quantified using substitutive standard substances. The calibration curves for the quantification of each saponin were selected and listed in Table 2. The developed analytical method was successfully applied to analysis of ten batches of the XST injection. All of the 27 characteristic peaks were determined simultaneously and the results are in Table 3. In the XST injection, the content of ginsenoside Rb_1 _was the most (26.17%-29.60%), followed by ginsenoside Rg_1 _(20.50%-25.43%), Rd (6.82%-8.10%), notoginsenoside R_1 _(5.29%-6.89%) and ginsenoside Re (2.91%-4.92%). The total content of the five saponins made up 61.69%-71.39% of the total saponins in the XST injection (total saponins nominal: 50 mg/ml). The ten saponins with available standard substances were quantitatively determined and made up 68.46%-75.85% of the total saponins nominal. Thus, combined with the semi-quantification data, 81.81%-95.71% components in the XST injection could be examined.

**Table 2:** Calibration curves, detection limits and quantification limits of the saponins by HPLC-UV

**Peak No**.	Saponins	**M.W**.	**Calibration curve **^**a**^	Linear range (μg/ml)	***R***^***2***^	LOD (μg/ml)
21	Ginsenoside Rk_3_	619	y = 6.7519x - 7.6085			
22	Ginsenoside Rh_4_	619	y = 6.7519x - 7.6085			
12	Ginsenoside Rh_1_	637	y = 6.7519x - 7.6085	4.28-68.5	0.9993	2.14
14	Ginsenoside F_1_	637	y = 6.7519x - 7.6085			

17	Notoginsenoside T_5_/Unkown	752	y = 5.4845x - 4.8387			
19	Notoginsenoside T_5_/Unkown	752	y = 5.4845x - 4.8387			
18	Unkown	765	y = 5.4845x - 4.8387			
20	Unkown	765	y = 5.4845x - 4.8387			
26	Ginsenoside Rk_1_	765	y = 5.4845x - 4.8387			
27	Ginsenoside Rg_5_	765	y = 5.4845x - 4.8387			
11	Ginsenoside Rg_2_	783	y = 5.6715x - 5.6679	3.34-53.5	0.9993	1.67
23	20(S)-Rg_3_	783	y = 5.4845x - 4.8387	2.95-47.3	0.9990	1.48
24	20(R)-Rg_3_	783	y = 5.0923x - 2.8995	2.63-42.0	0.9994	1.75
25	Ginsenoside F_2_	783	y = 5.4845x - 4.8387			

2	Ginsenoside Rg_1_	800	y = 5.1367x - 76.471	16.64-1065	0.9990	10.29
5	Ginsenoside Rf	800	y = 5.1367x - 76.471			

8	SC1	901	y = 4.3254x - 5.0843			
1	Notoginsenoside R_1_	932	y = 4.3254x - 5.0843	10.26-492.5	0.9997	7.42

3	Ginsenoside Re	945	y = 4.4123x - 29.465	43.28-692.5	0.9993	4.73
15	Ginsenoside Rd	945	y = 4.1199x - 5.5681	16.64-532.5	0.9993	4.43
16	Notoginsenoside K	945	y = 4.1199x - 5.5681			

13	Ginsenoside Rb_2_	1077	y = 3.8757x + 2.4182	4.84-77.5	0.9995	1.95
7	Notoginsenoside I	1092	y = 3.8757x + 2.4182			

9	Ginsenoside Rb_1_	1107	y = 3.5815x - 29.548	15.98-1022.5	0.9992	7.91
10	Notoginsenoside Fc	1209	y = 3.5815x - 29.548			
4	Notoginsenoside R_4_	1240	y = 3.5815x - 29.548			
6	Notoginsenoside Fa	1240	y = 3.5815x - 29.548			

^a ^y: peak area of analyte; x: concentration of analyte (μg/ml)

**Table 3:** Contents (%) of the 27 saponins in the XST injection (total saponins nominal: 50 mg/ml) ^a^

**Peak No**.	Saponins	S1	S2	S3	S4	S5	S6	S7	S8	S9	S10
1	Notoginsenoside R_1 _(%)	6.64	5.29	6.89	6.47	6.27	5.86	5.33	6.41	6.07	6.35
2	Ginsenoside Rg_1 _(%)	25.43	20.50	24.53	23.99	23.76	20.29	21.15	22.23	22.31	23.33
3	Ginsenoside Re (%)	3.43	2.91	4.92	3.61	3.55	3.56	3.35	3.04	3.03	3.69
4	Notoginsenoside R_4 _(%)	1.52	1.19	1.24	1.33	1.28	1.33	1.31	1.11	1.15	1.38
5	Ginsenoside Rf (%)	1.24	0.95	0.98	1.15	1.15	0.97	1.03	1.03	1.03	1.00
6	Notoginsenoside Fa (%)	1.45	1.21	1.90	1.35	1.44	1.43	1.35	1.29	1.29	1.34
7	Notoginsenoside I (%)	0.89	0.62	0.17	0.80	0.80	0.76	0.81	0.73	0.66	0.83
8	SC1 (%)	0.65	0.51	2.28	0.56	0.62	0.46	0.54	0.52	0.49	0.54
9	Ginsenoside Rb_1 _(%)	28.39	26.17	26.34	28.30	28.78	29.58	29.60	28.00	28.14	27.78
10	Notoginsenoside Fc (%)	1.30	0.94	0.99	1.13	1.12	1.06	0.98	1.05	1.05	1.15
11	Ginsenoside Rg_2 _(%)	1.02	1.31	1.08	1.18	0.98	0.78	1.44	1.38	1.38	1.17
12	Ginsenoside Rh_1 _(%)	1.77	3.06	2.25	2.22	1.65	1.06	2.90	3.19	3.22	2.17
13	Ginsenoside Rb_2 _(%)	1.09	0.69	2.18	1.07	1.06	1.00	0.90	0.81	1.11	1.04
14	Ginsenoside F_1 _(%)	0.76	1.77	0.29	1.14	0.85	0.50	1.59	1.90	1.88	1.24
15	Ginsenoside Rd (%)	7.50	6.82	7.25	7.22	7.24	7.27	8.10	7.41	7.48	7.18
16	Notoginsenoside K (%)	1.01	0.72	1.05	1.18	1.24	1.33	1.36	0.96	1.04	1.43
17	Notoginsenoside T_5_/Unkown (%)	0.39	0.69	0.58	0.69	0.47	0.39	0.79	0.87	0.86	0.83
18	Unkown (%)	0.30	0.37	1.11	0.45	0.36	0.23	0.56	0.50	0.50	0.46
19	Notoginsenoside T_5_/Unkown (%)	0.72	1.31	0.41	1.19	0.82	0.63	1.51	1.51	1.54	1.20
20	Unkown (%)	0.39	0.55	0.31	0.55	0.37	0.39	0.70	0.66	0.67	0.55
21	Ginsenoside Rk_3 _(%)	0.90	2.30	1.59	1.78	1.10	0.80	2.35	2.52	2.57	1.77
22	Ginsenoside Rh_4 _(%)	1.27	3.66	2.47	2.69	1.49	0.91	3.70	3.87	3.88	2.65
23	20S-Rg_3 _(%)	0.37	1.01	0.75	0.81	0.44	0.43	1.21	1.09	1.14	0.83
24	20R-Rg_3 _(%)	0.21	0.70	0.52	0.51	0.25	0.22	0.78	0.76	0.82	0.56
25	Ginsenoside F_2 _(%)	0.36	0.38	0.23	0.28	0.14	0.10	0.78	0.42	0.43	0.25
26	Ginsenoside Rk_1 _(%)	0.41	1.13	1.22	0.81	0.66	0.47	1.62	1.02	1.28	0.80
27	Ginsenoside Rg_5 _(%)	0.32	1.30	1.17	1.05	0.65	0.46	1.95	1.31	1.50	1.03
	**Total **(%) ^b^	89.41	86.78	93.54	92.47	87.90	81.81	95.71	94.27	95.02	91.50

^a ^Mean values of samples (*n *= 3)

^b ^Total content of the 27 saponins in samples

## Conclusion

The fingerprint profiles of ten batches of samples showed 27 characteristic peaks. Ten of these 27 saponins in the XST injections were quantitatively determined with their standard references; the rest 17 saponins were semi-quantified with the substitutive standard references.

## Abbreviations

XST: Xuesaitong; HPLC-UV: high performance liquid chromatography-ultraviolet detection; HPLC-PDA/ESI-MS^n^: HPLC with photo diode array detection/electrospray ionization tandem mass spectrometry; HPLC-ELSD: high performance liquid chromatography-evaporative light scattering detection; HPLC-MS: high performance liquid chromatography-mass spectroscopy; SFDA: State Food and Drug Administration (China)

## Competing interests

The authors declare that they have no competing interests.

## Authors' contributions

XHF designed the study. HY performed the fingerprint and quantitative analysis and wrote the manuscript. PYS and QS assisted HY to identify the characteristic peaks using HPLC-PDA/ESI-MS^n^. All authors read and approved the final version of the manuscript.

## Supplementary Material

Additional file 1 **The chromatogram of similarity analysis of the fingerprints of 10 samples**. Click here for file

Additional file 2 **The similarities of chromatograms of 10 samples (n = 3)**. Click here for file

Additional file 3 **PDA Chromatograms**. standard compounds (A) and a XST injection (C), and total ion current chromatograms of standard compounds (B) and a XST injection (D). 1-27 were the characteristic peaks, listed in Table 2 Click here for file

Additional file 4 **Plots of slopes of calibration curves vs. molecular weights (MW) of saponins**. From literatures (A) [Journal of Pharmaceutical and Biomedical Analysis 41 (2006) 274-279], (B) [Journal of Pharmaceutical and Biomedical Analysis 48 (2008) 1361-1367], (C) [Journal of Pharmaceutical and Biomedical Analysis 38 (2005) 45-51], (D) [Journal of Chromatography A 1011 (2003) 77-87], (E) [Journal of Shenyang Pharmaceutical University Vol. 20, No.1 (2003) 27-31], and (F) [Chinese Pharmaceutical Journal Vol. 38, No.9 (2003) 698-699] Click here for file

Additional file 5 **The method validation for simultaneous determination of the twenty-seven saponins in XST injection**. The quantitative and semi-quantitative methods were validated and the semi-quantitative principle were discussed in detail. Click here for file

Additional file 6 **Precisions and repeatability**. The results of precision and repeatability for simultaneous determination of the twenty-seven saponins Click here for file

Additional file 7 **Recovery**. The results of recovery for simultaneous determination of the twenty-seven saponins Click here for file

Additional file 8 **Plots of slopes of calibration curves vs molecular weights (MW) with different chromatography columns**. (A) Ultimate™ XB-C18 (250 mm × 4.6 mm, 5 μm), (B) Zorbax Eclipse SB-C18 (250 mm × 4.6 mm, 5 μm) and (C) Zorbax Eclipse SB-C18 (100 mm × 2.1 mm, 1.8 μm) Click here for file

Additional file 9 **Regression equation using different columns**. Columns: Zorbax Eclipse SB-C18 (250 mm × 4.6 mm, 5 μm) and Zorbax Eclipse SB-C18 (100 mm × 2.1 mm, 1.8 μm) Click here for file
