# Supplementary material for: Chemical fingerprinting and quantitative analysis of a Panax notoginseng preparation using HPLC-UV and HPLC-MS
Source: Chin Med. 2011 Feb 24;6:9. doi: 10.1186/1749-8546-6-9 (PMC3052241; doi:10.1186/1749-8546-6-9)
Supplement: Additional file 1 — The chromatogram of similarity analysis of the fingerprints of 10 samples. [file 1749-8546-6-9-S1.PDF]

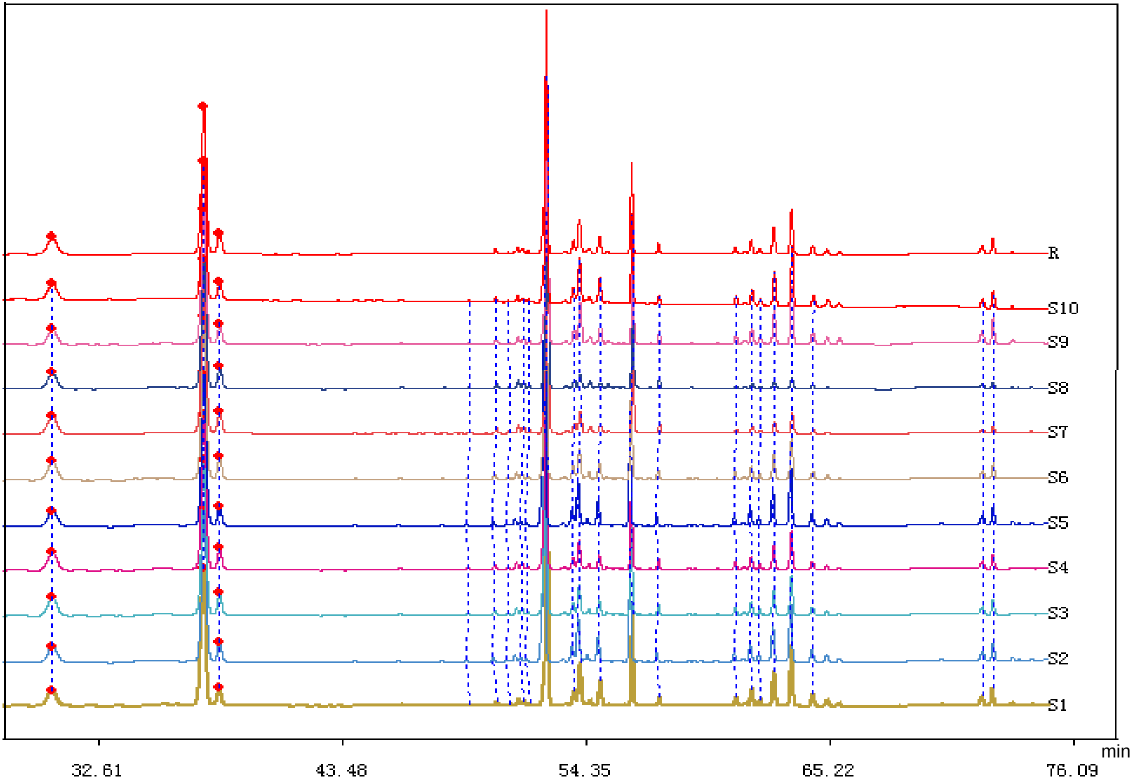

Chromatogram of similarity analysis of the fingerprints of 10 samples Similarity Evaluation software.
