# Supplementary material for: Chemical fingerprinting and quantitative analysis of a Panax notoginseng preparation using HPLC-UV and HPLC-MS
Source: Chin Med. 2011 Feb 24;6:9. doi: 10.1186/1749-8546-6-9 (PMC3052241; doi:10.1186/1749-8546-6-9)
Supplement: Additional file 2 — The similarities of chromatograms of 10 samples (n = 3). [file 1749-8546-6-9-S2.PDF]

The similarities of chromatograms of 10 samples (n=3)

Samples	Similarities (mean ±SD)
**S1**	0.996 ± 0.002
**S2**	0.989 ± 0.001
**S3**	0.999 ± 0.002
**S4**	0.999 ± 0.001
**S5**	0.996 ± 0.001
**S6**	0.988 ± 0.001
**S7**	0.996 ± 0.002
**S8**	0.995 ± 0.001
**S9**	0.998 ± 0.001
**S10**	0.999 ± 0.001
**Reference fingerprint**	1
