# Supplementary material for: Chemical fingerprinting and quantitative analysis of a Panax notoginseng preparation using HPLC-UV and HPLC-MS
Source: Chin Med. 2011 Feb 24;6:9. doi: 10.1186/1749-8546-6-9 (PMC3052241; doi:10.1186/1749-8546-6-9)
Supplement: Additional file 3 — PDA Chromatograms. standard compounds (A) and a XST injection (C), and total ion current chromatograms of standard compounds (B) and a XST injection (D). 1-27 were the characteristic peaks, listed in Table 2 [file 1749-8546-6-9-S3.PDF]

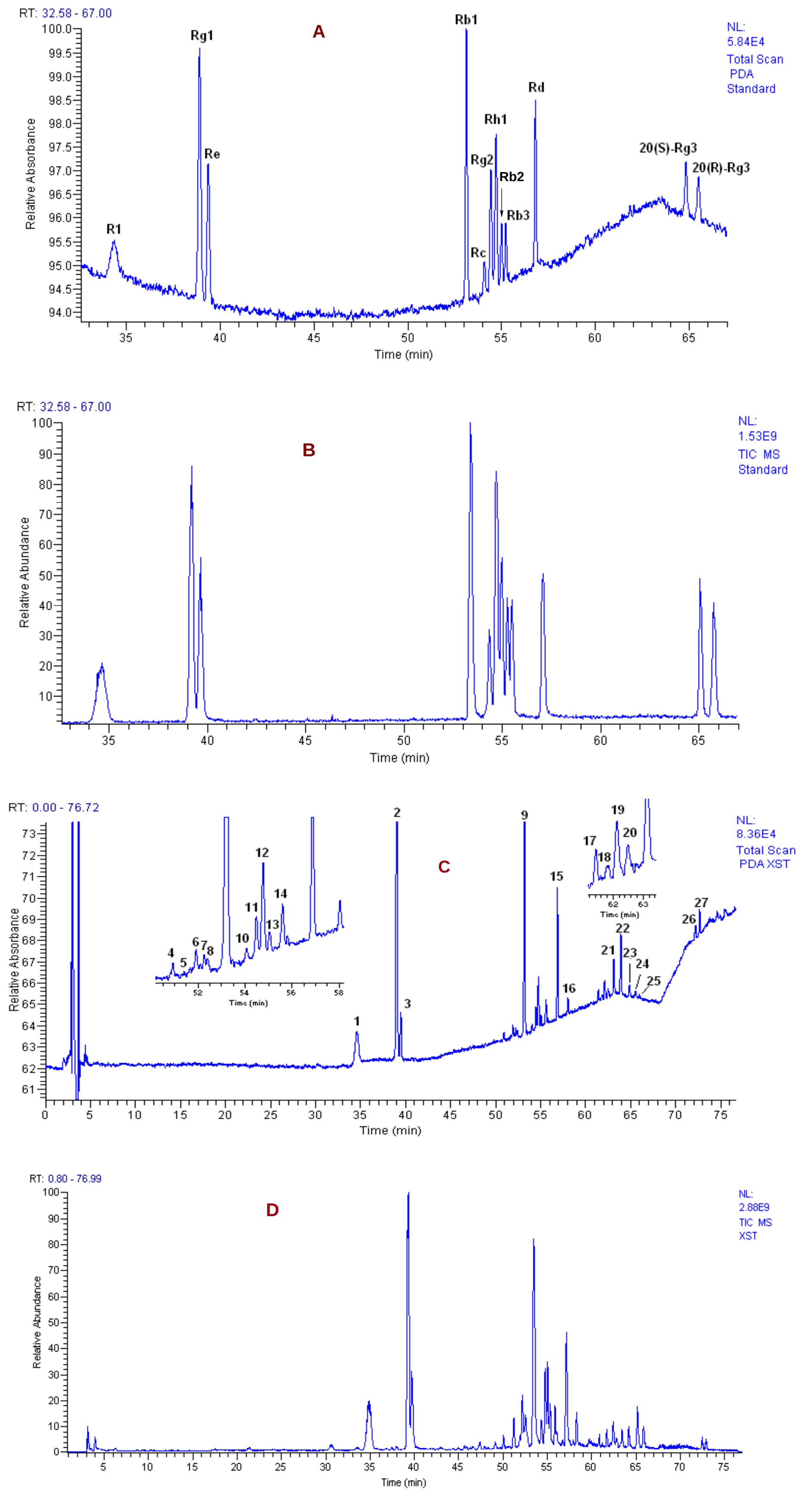

PDA Chromatograms of standard compounds (A) and a XST injection (C), and total ion current chromatograms of standard compounds (B) and a XST injection (D). 1–27 were the characteristic peaks, listed in Table 2.
