# Supplementary material for: Chemical fingerprinting and quantitative analysis of a Panax notoginseng preparation using HPLC-UV and HPLC-MS
Source: Chin Med. 2011 Feb 24;6:9. doi: 10.1186/1749-8546-6-9 (PMC3052241; doi:10.1186/1749-8546-6-9)
Supplement: Additional file 4 — Plots of slopes of calibration curves vs. molecular weights (MW) of saponins. From literatures (A) [Journal of Pharmaceutical and Biomedical Analysis 41 (2006) 274-279], (B) [Journal of Pharmaceutical and Biomedical Analysis 48 (2008) 1361-1367], (C) [Journal of Pharmaceutical and Biomedical Analysis 38 (2005) 45-51], (D) [Journal of Chromatography A 1011 (2003) 77-87], (E) [Journal of Shenyang Pharmaceutical University Vol. 20, No.1 (2003) 27-31], and (F) [Chinese Pharmaceutical Journal Vol. 38, No.9 (2003) 698-699] [file 1749-8546-6-9-S4.PDF]

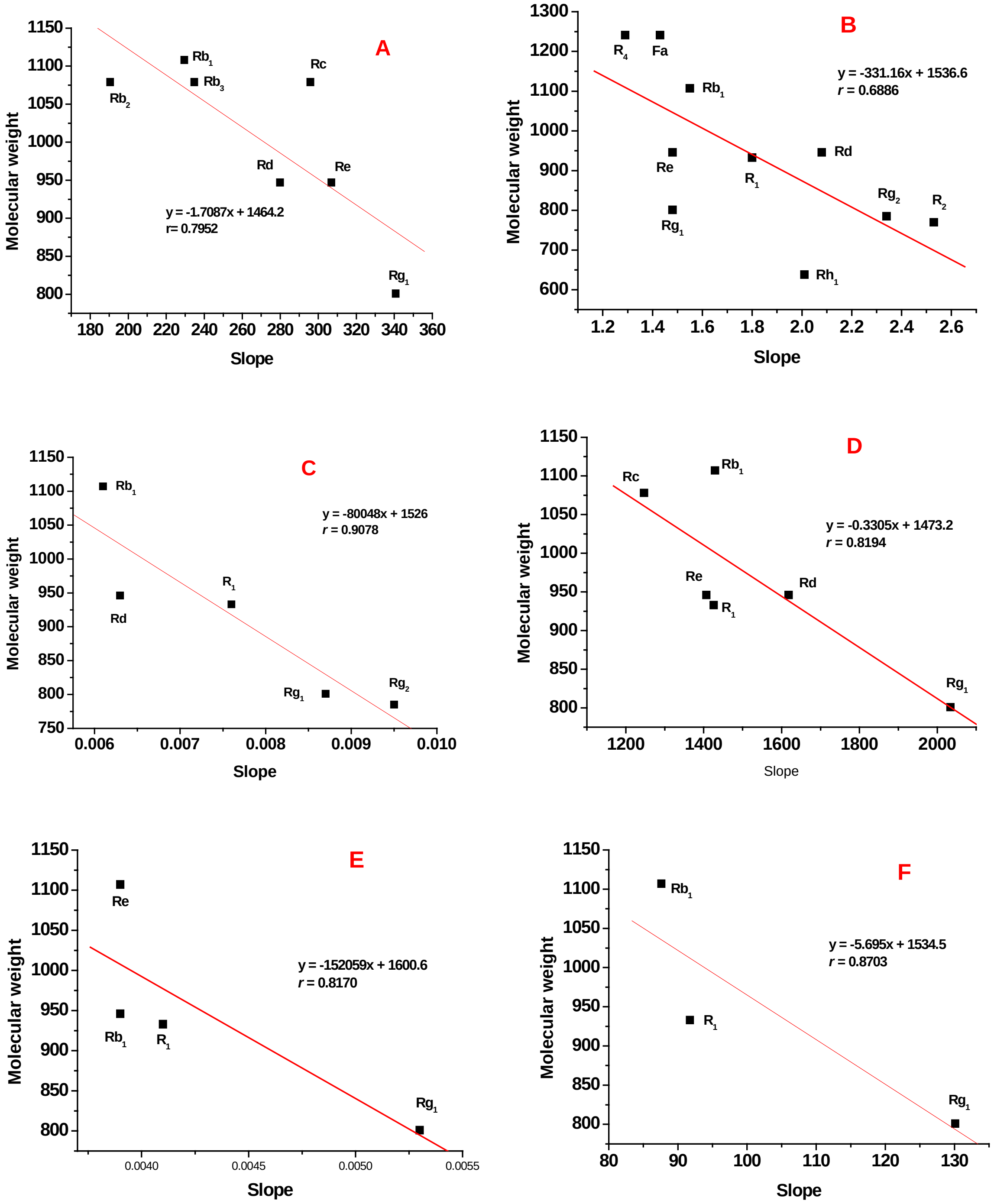

Plots of slopes of calibration curves vs. molecular weights (MW) of saponins from literatures (A) [Journal of Pharmaceutical and Biomedical Analysis 41 (2006) 274–279], (B) [Journal of Pharmaceutical and Biomedical Analysis 48 (2008) 1361–1367], (C) [Journal of Pharmaceutical and Biomedical Analysis 38 (2005) 45–51], (D) [Journal of Chromatography A 1011 (2003) 77–87], (E) [Journal of Shenyang Pharmaceutical University Vol. 20, No.1 (2003) 27–31], and (F) [Chinese Pharmaceutical Journal Vol. 38, No.9 (2003) 698–699]
