# Supplementary material for: Chemical fingerprinting and quantitative analysis of a Panax notoginseng preparation using HPLC-UV and HPLC-MS
Source: Chin Med. 2011 Feb 24;6:9. doi: 10.1186/1749-8546-6-9 (PMC3052241; doi:10.1186/1749-8546-6-9)
Supplement: Additional file 5 — The method validation for simultaneous determination of the twenty-seven saponins in XST injection. The quantitative and semi-quantitative methods were validated and the semi-quantitative principle were discussed in detail. [file 1749-8546-6-9-S5.DOC]

These interesting findings inspired us that perhaps all of the 27 saponins in XST injections would be able to be quantified with the 10 standard substances above mentioned, if it could be found the substitutive calibration curves for saponins without standard compounds from those of 10 standard substances. For this goal, the method validation was performed as follows.

At first, the method was validated with standard solutions in terms of linearity, limits of detection (LODs), precision, repeatability and recovery test.

A mixed stock solution consisting of notoginsenoside R_1_ (1.970mg/ml), ginsenoside Rg_1_ (4.260mg/ml), Re (2.770mg/ml), Rb_1_ (4.090mg/ml), Rg_2_ (0.214mg/ml), Rh_1_ (0.274mg/ml), Rb_2_ (0.310mg/ml), Rd (2.130mg/ml), 20(S)-Rg_3_ (0.189mg/ml) and 20(R)-Rg_3_ (0.168mg/ml), was prepared. A series of standard working solution with eight different concentration levels was prepared by diluting appropriately the mixed stock solution with 80% aqueous methanol (v/v) for the standard curves. An aliquot (10μl) of each standard working solution was subject to HPLC-UV analysis. The linearity for each compound was established by plotting the peak area (*y*) versus the concentration (*x*) of each analyte, which was demonstrated by the equation listed in Table 2. All calibration curves showed good linearity (R^2^>0.999) within test ranges. The LODs were determined at signal-to-noise ratios (S/N) of 3. Standard solution containing ten reference compounds was diluted to a series of appropriate concentrations with 80% aqueous methanol (v/v) for HPLC-UV analysis. As shown in Table 2, the LODs for the analytes were less than 11μg/ml.

Intra- and inter-day precisions were determined by analyzing the replicate samples (batch No: 20081018) within one day and on three consecutive days, respectively. The results were expressed with relative standard deviations (RSD) of areas of the 27 characteristic peaks. For the repeatability test, five different working solutions prepared from the same sample (batch No: 20081018) were analyzed. The variations of areas of the 27 characteristic peaks were calculated as repeatability results. The RSD values of the intra- and inter-day precisions were less than 8.6%, and the repeatability was less than 9.1% for the analytes (Additional file 6).

The recovery test was determined by standard addition method. The ten saponins were spiked into the injection samples (batch No: 20081018), and then diluted and quantified in accordance with the procedures described earlier. The results were summarized in Supplementary Additional file 7. The recoveries of the ten saponins were 92.8–104.6%, and their RSD values were less than 9.1%. Therefore, the HPLC-UV method was precise, accurate, and sensitive enough for simultaneous determination of the 10 saponins in the XST injection.

From Table 2, indeed, it could be found that slopes of calibration curves for the ten reference saponins were negative relative to their molecular weights by HPLC-UV at 203nm (Additional file 8), and the regression equations of saponins with similar or same molecular weights were also close to each other. Furthermore, another two Zorbax Eclipse SB-C_18_ columns (250mm×4.6mm, 5μm; 100mm×2.1mm, 1.8μm) were also used to determine the reference saponins to verify the findings above-mentioned (the regression equations are shown in Additional file 9. The approximate negative correlation relationship between molecular weights of saponins and the slopes of calibration curves was also observed using the different chromatography columns. Moreover, the regression equations for notoginsenoside R_1_, ginsenoside Re, and Rd with similar or equal molecular weights (933, 947 and 947 respectively) were close to each other, and so did that for ginsenoside 20(S)-Rg_3_, 20(R)-Rg_3_ and Rg_1_ (785, 785 and 801 respectively). These results suggested that the findings were regular. Actually, the observations may be explained reasonably. As it known, these saponins have similar structures, which could cause them possessing similar molar absorptivity at 203nm. With the concentration of saponins increasing one unit (∆C=1μg/ml), the increased molecular number (increased light extinction particles) for the saponin with a higher molecular weight would be less than that for the saponin with a lower molecular weight in chromatography, which could also result in the increment amplitude of the peak area (∆A) for the higher molecular weight saponin in chromatography to be less comparing to the ∆A for the lower molecular weight saponins (owing to that the peak area remains proportional to the total molecular number of substance passing into the detector). Accordingly, it is understandable to that the slopes of calibration curves for the saponins with similar structure characteristics were approximately negative relative to their molecular weights by HPLC-UV at 203nm.

Thus, according to the discussion above, we can select substitutive standard substances for the saponins without standard references from the available standard saponins according to their molecular weights. Even, we can quantify the saponins in XST injections by the substitutive standard substances without knowing their exact molecular structure. For example, in Figure 2A, peak 17 (notoginsenoside T_5_/Unkown), 18 (unknown), 19 (notoginsenoside T_5_/unkown), 20 (unknown), 26 (ginsenoside Rk_1_), 27 (ginsenoside Rg_5_) have similar or same molecular weight to that of ginsenoside Rg_2_, or 20(S)-Rg_3_ and 20(R)-Rg_3_ (all the molecular weight information can be obtained from the HPLC-ESI-MS^n^ data). Therefore, these saponins can be semi-quantified with calibration curve of ginsenoside Rg_2_, or 20(S)-Rg_3_ and 20(R)-Rg_3_. Furthermore, no matter which was selected from calibration curves of the three standard saponins as the substitutive one, the calculated results were very similar to each other (the relative standard deviation of the results is less than 5% when the concentration of the analyte is set at 3μg/ml). In order to further validate the semi-quantified method, the recovery test for 20(R)-Rg_3_ was also evaluated by using the calibration curve for 20(S)-Rg_3_ as the substitutive one for 20(R)-Rg_3_. As shown in Additional file 6, the calculated average recovery and the relative standard deviation of the resulting for 20(R)-Rg_3_ were 99.0% and 7.4% respectively, suggesting that the presented semi-quantified method could work well for the assay of the saponins without standard references in the XST injection.
