# Supplementary material for: Chemical fingerprinting and quantitative analysis of a Panax notoginseng preparation using HPLC-UV and HPLC-MS
Source: Chin Med. 2011 Feb 24;6:9. doi: 10.1186/1749-8546-6-9 (PMC3052241; doi:10.1186/1749-8546-6-9)
Supplement: Additional file 6 — Precisions and repeatability. The results of precision and repeatability for simultaneous determination of the twenty-seven saponins [file 1749-8546-6-9-S6.PDF]

Precisions and repeatability

Peak No.	Saponins	Intra-day Precision (%)	Inter-day Precision (%)	Repeatability
1	Notoginsenoside R_1	2.2	3.4	2.4
2	Ginsenoside Rg_1	1.0	1.4	0.6
3	Ginsenoside Re	7.7	5.1	0.88
4	Notoginsenoside R_4	2.2	4.1	3.6
5	Ginsenoside Rf	2.7	0.5	5.4
6	Notoginsenoside Fa	1.9	5.9	2.5
7	Notoginsenoside I	2.3	3.6	2.3
8	SC1	0.9	3.3	3.4
9	Ginsenoside Rb_1	0.5	2.9	1.1
10	Notoginsenoside Fc	2.7	3.3	5.1
11	Ginsenoside Rg_2	1.3	2.8	3.3
12	Ginsenoside Rh_1	0.7	2.8	2.1
13	Ginsenoside Rb_2	0.9	1.6	5.4
14	Ginsenoside F_1	3.3	2.6	7.9
15	Ginsenoside Rd	1.4	3.8	0.7
16	Notoginsenoside K	1.0	1.0	2.9
17	Notoginsenoside T_5/Unkown	3.7	0.8	9.1
18	Unkown	0.7	4.6	7.3
19	Notoginsenoside T_5/Unkown	3.5	5.5	2.4
20	Unkown	1.8	2.9	7.7
21	Ginsenoside Rk_3	2.2	3.3	2.4
22	Ginsenoside Rh_4	0.7	3.6	2.2
23	20(S)-Rg_3	2.1	2.2	3.0
24	20(R)-Rg_3	1.9	2.4	4.7
25	Ginsenoside F_2	6.3	8.6	3.8
26	Ginsenoside Rk_1	2.6	3.0	4.5
27	Ginsenoside Rg_5	5.9	3.4	4.4
