# Supplementary material for: Chemical fingerprinting and quantitative analysis of a Panax notoginseng preparation using HPLC-UV and HPLC-MS
Source: Chin Med. 2011 Feb 24;6:9. doi: 10.1186/1749-8546-6-9 (PMC3052241; doi:10.1186/1749-8546-6-9)
Supplement: Additional file 7 — Recovery. The results of recovery for simultaneous determination of the twenty-seven saponins [file 1749-8546-6-9-S7.PDF]

Recovery

**Analytes**	**Added (μg/ml)**	**found(μg/ml, n=6)**	**Recovery (%, n=6)**	**RSD (%, n=6)**
Notoginsenoside R_1	237.50	239.96	101.0	4.7
Ginsenoside Rg_1	658.75	660.75	100.3	7.3
Ginsenoside Re	302.50	325.44	107.6	3.2
Ginsenoside Rb_1	506.25	505.05	99.8	4.5
Ginsenoside Rg_2	25.00	23.92	95.7	4.0
Ginsenoside Rh_1	33.75	31.97	94.7	1.4
Ginsenoside Rb_2	31.25	28.94	92.6	2.6
Ginsenoside Rd	283.75	296.23	104.4	3.2
Ginsenoside 20(S)-Rg_3	23.63	24.76	104.8	3.4
Ginsenoside 20(R)-Rg_3	7.00	7.08	101.1	9.1
Ginsenoside 20(R)-Rg_3 *	7.00	6.93	99.0	7.4

* The recovery and RSD results were obtained by using the calibration curve for 20(S)-Rg_3 as the substitutive one for 20(R)-Rg_3.
