# Supplementary material for: Chemical fingerprinting and quantitative analysis of a Panax notoginseng preparation using HPLC-UV and HPLC-MS
Source: Chin Med. 2011 Feb 24;6:9. doi: 10.1186/1749-8546-6-9 (PMC3052241; doi:10.1186/1749-8546-6-9)
Supplement: Additional file 8 — Plots of slopes of calibration curves vs molecular weights (MW) with different chromatography columns. (A) Ultimate™ XB-C18 (250 mm × 4.6 mm, 5 μm), (B) Zorbax Eclipse SB-C18 (250 mm × 4.6 mm, 5 μm) and (C) Zorbax Eclipse SB-C18 (100 mm × 2.1 mm, 1.8 μm) [file 1749-8546-6-9-S8.PDF]

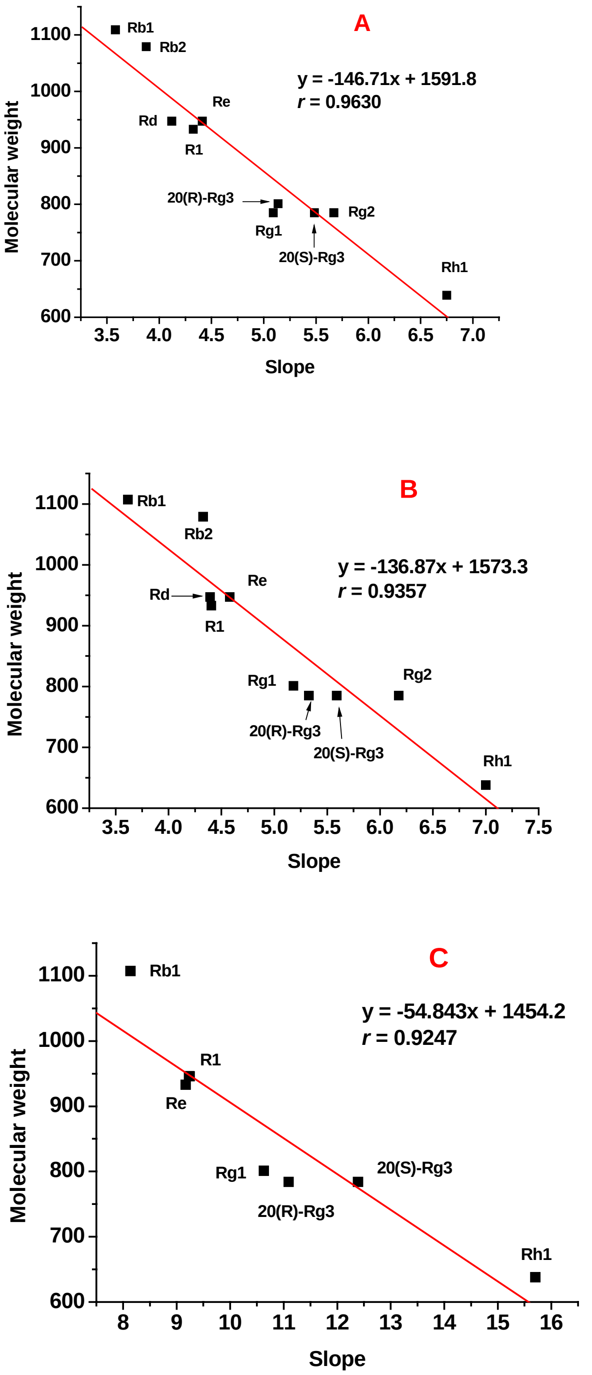

Plots of slopes of calibration curves vs molecular weights (MW) with chromatography columns (A) Ultimate™ XB-C_{18} (250 mm × 4.6 mm, 5 μm) , (B) Zorbax Eclipse SB-C_{18} (250 mm × 4.6 mm, 5 μm) and (C) Zorbax Eclipse SB-C_{18} (100 mm × 2.1 mm, 1.8 μm)
