# Supplementary material for: Chemical fingerprinting and quantitative analysis of a Panax notoginseng preparation using HPLC-UV and HPLC-MS
Source: Chin Med. 2011 Feb 24;6:9. doi: 10.1186/1749-8546-6-9 (PMC3052241; doi:10.1186/1749-8546-6-9)
Supplement: Additional file 9 — Regression equation using different columns. Columns: Zorbax Eclipse SB-C18 (250 mm × 4.6 mm, 5 μm) and Zorbax Eclipse SB-C18 (100 mm × 2.1 mm, 1.8 μm) [file 1749-8546-6-9-S9.PDF]

Regression equations using different columns

**Analytes**	**Molecular weight**	**Eclipse SB-C_{18} columns (250 mm × 4.6 mm, 5 μm)**	**Eclipse SB-C_{18} columns (100 mm × 2.1 mm, 1.8 μm)**
Notoginsenoside R_1	933	$y = 4.4048x + 2.9856$ ($R^2 = 0.9991$)	$y = 9.1699x + 47.877$ ($R^2 = 0.9996$)
Ginsenoside Rg_1	801	$y = 5.1815x - 20.394$ ($R^2 = 0.9994$)	$y = 10.963x + 131.02$ ($R^2 = 0.9996$)
Ginsenoside Re	947	$y = 4.5804x + 30.827$ ($R^2 = 0.9997$)	$y = 9.2456x + 157.68$ ($R^2 = 0.9985$)
Ginsenoside Rb_1	1109	$y = 3.617x + 14.933$ ($R^2 = 0.9993$)	$y = 8.1382x + 111.42$ ($R^2 = 0.9983$)
Ginsenoside Rg_2	785	$y = 6.177x - 1.9277$ ($R^2 = 0.9992$)	–
Ginsenoside Rh_1	621	$y = 7.0041x - 1.4248$ ($R^2 = 0.9994$)	$y = 15.702x - 26.373$ ($R^2 = 0.9994$)
Ginsenoside Rb_2	1079	$y = 4.3273x - 1.5284$ ($R^2 = 0.9995$)	–
Ginsenoside Rd	947	$y = 4.3922x + 7.5718$ ($R^2 = 0.9995$)	$y = 9.2312x + 86.574$ ($R^2 = 0.9985$)
Ginsenoside 20(S)-Rg_3	785	$y = 5.5903x - 2.0428$ ($R^2 = 0.9994$)	$y = 11.096x - 97.326$ ($R^2 = 0.9994$)
Ginsenoside 20(R)-Rg_3	785	$y = 5.3286x - 1.939$ ($R^2 = 0.9995$)	$y = 12.391x - 116.91$ ($R^2 = 0.9967$)

"–" means that this standard saponin is not added into the mixture standard solution.
